# The Atlanta VA Health Care System: Morehouse School of Medicine Core Recruiting Site—A Strategy to Increase Diversity in the VA Scientific Workforce

**DOI:** 10.1089/heq.2023.0001

**Published:** 2023-05-26

**Authors:** C. Michael Hart, Kennie R. Shepherd, Walter Royal

**Affiliations:** ^1^ Office of Research Administration, Atlanta VA Health Care System, Decatur, Georgia, USA.; ^2^ Department of Medicine, Emory School of Medicine, Atlanta, Georgia, USA.; ^3^ Department of Pharmacology and Toxicology, Morehouse School of Medicine, Atlanta, Georgia, USA.; ^4^ Department of Neurobiology and Director, Neuroscience Institute, Morehouse School of Medicine, Atlanta, Georgia, USA.

**Keywords:** health care, health disparities, diversity, Veterans

## Abstract

The Department of Veterans Affairs (VA) initiative to enhance recruitment of diverse biomedical scientists from Historically Black Colleges and Universities (HBCUs) through the VA Career Development Program has provided a unique opportunity for HBCUs to partner with VA to strengthen diversity recruitment efforts. The Atlanta VA Health Care System and the Morehouse School of Medicine (MSM) enjoy a productive and growing interinstitutional collaboration. The partnership between the Atlanta VA and MSM provides the unique opportunity for MSM to increase research opportunities for faculty and students while providing a pipeline of diverse candidates for the Atlanta VA to enhance recruitment of diverse HCBU biomedical scientists. This relationship led to the creation of an inaugural HBCU Core Recruitment Site (CRS) at MSM and the Atlanta VA. The CRS provides a pathway to identify and recruit young diverse investigators who are eligible to compete for VA Career Development Award funding. This Atlanta VA/MSM CRS initiative established a pipeline program to further enhance diversity in the VA scientific workforce. In this review, the Atlanta VA/MSM CRS is presented as a potential model for maximizing the VA initiative to enhance the recruitment of diverse candidates from HBCUs.

## Introduction

In October 2019, the Department of Veterans Affairs (VA) Office of Research and Development (ORD) announced a unique funding opportunity designed to leverage existing relationships between VA Health Care facilities and Historically Black Colleges and Universities (HBCUs). This program sought to establish HBCU Core Recruiting Sites (CRS) that would facilitate the recruitment of candidates from HBCUs to apply for VA Career Development Awards (CDA). As these CDA awards provide eligible candidates with salary, research support, and mentored training for 3–5 years, they generally target recruitment of individuals who are completing doctoral degrees in scientific disciplines, postdoctoral fellowships, or medical residency or fellowship training.

VA CDAs are designed to attract and train early career academic faculty to fields related to Veterans' health. This program also assists VA facilities and their academic affiliates in the recruitment, training, and retention of high-quality clinician and nonclinician scientists within the VA health care system. The CRS funding mechanism provided a new opportunity to grow and tighten the affiliation between HBCU institutions and VA facilities proximate to their location, to accelerate the diversification of the VA scientific workforce, and to enhance awareness among talented HBCU trainees of professional opportunities in VA research.

Starting in 2005, the Atlanta VA Health Care System shared formal educational affiliations with Morehouse School of Medicine (MSM). At that time, the nature of this relationship was predominantly focused on clinical training opportunities for Morehouse Graduate Medical Education trainees at VA. Many VA faculty members held adjunct faculty appointments at MSM to support these training activities. Morehouse had also played a prominent role in the development of the Women's Center of Excellence located at the Fort McPherson VA Clinic in Atlanta. This affiliation between the Atlanta VA and MSM demonstrated functional and robust interinstitutional interactions at an educational level. The Atlanta VA and MSM were further affiliated through their mutual participation in the NIH-sponsored Georgia Clinical and Translational Science Alliance based at Emory University in Atlanta.

Although sporadic research collaborations between VA and Morehouse investigators had occurred, few Morehouse faculties had applied for VA funding and awareness of shared research and collaborative opportunities between the scientific faculty of MSM and the Atlanta VA was limited. The opportunity to further develop this collaboration was strengthened in June 2018 when MSM recruited Dr. Walter Royal III, an accomplished neurologist and physician-scientist, with an established VA scientific program funded by a VA Merit Review Award. As professor and chair of neurobiology at MSM and director of the Morehouse Neuroscience Institute, Dr. Royal provided a clear example for faculty success at the Atlanta VA and MSM in the combined practice of medicine and VA-funded research program(s) at both sites.

Leaders at the Atlanta VA and MSM recognized their unique opportunity in Atlanta to respond to the ORD solicitation to establish a HBCU CRS within VA. This objective aligned well with local Atlanta VA strategic initiatives at that time to grow their research program in part through the CDA mechanism. Based on awareness that rigorous research experiences coupled with effective mentoring can enhance biomedical investigator diversity,^1^ leaders in research at the Atlanta VA and at MSM jointly considered this collaborative opportunity and elected to proceed with an application.

As the CRS funding mechanism was designed to provide institutional support for recruitment activities and programs, it allowed applicant institutions significant flexibility and creativity in the structure and design of their program. Central assumptions in the funding solicitation described “the establishment of a sustainable, continuous pipeline of early-career researchers through outreach and information sharing with the [HBCU] affiliate.” The Atlanta VA-MSM CRS was developed with intention to respond to these broad guidelines. In this article, we describe our experience to date as a VA ORD-funded CRS and the evolution of practice and ideas to achieve its objectives.

## CRS Program Characteristics

Initial assessments indicated that the MSM supported the training of a large number of under-represented minority candidates who were well qualified and prepared to enter the VA Career Development Program. In 2018, for example, the MSM had matriculated 78 students pursuing the Doctor of Medicine (MD) degree. The MSM PhD program in Biomedical Sciences also provided advanced research training as well as dual PhD/Master of Science in Clinical Research degrees to nearly 40 predoctoral students. More than 70% of the PhD programs' graduates had gone on to postdoctoral research fellowships or other advanced academic training. The number of MD and PhD trainees graduating from Morehouse had increased substantially over the previous decade providing a rich source of potential candidates for VA CDA training and awards.

Based on this large number of potential candidates, the CRS envisioned joint trainee mentorship by VA and Morehouse faculty. While applications focused on any scientific discipline relevant to Veterans' health would be welcomed from eligible Morehouse candidates, several areas of mutual institutional scientific strength were identified and felt to provide fertile opportunities for interinstitutional scientific mentorship committees, collaboration, and training. These broad areas of focus to be targeted by initial CRS efforts included Cancer Research, Cardiovascular Disease, Neurosciences, and Infectious Diseases, including HIV/AIDS.

The administrative structure of the CRS was developed collaboratively by its executive associate directors C. Michael Hart, MD, and Walter Royal III, MD, Atlanta VA Staff Physicians. As Drs. Hart and Royal were both VA Merit Review-funded investigators and senior clinicians with significant clinical and research training experience at VA, they were familiar with strategies to recruit CDA candidates. As the associate chief of staff for research for the Atlanta VA Healthcare System, Dr. Hart was able to provide the support of the Atlanta VA Research Office to the CRS program. VA research administrative personnel provide training, advice, and assistance to the CRS director and his assistant, and to Morehouse CDA applicants participating in this program.

A search was undertaken to identify a CRS director targeting existing MSM faculty who had knowledge of Morehouse programs and personnel. Kennie Shepherd, PhD, was identified by leadership at the MSM as an ideal candidate for the position. Dr. Shepherd serves as assistant dean for admissions and student affairs in MSM and is associate professor of pharmacology and toxicology at Morehouse. Dr. Shepherd brought to his position significant experience recruiting under-represented minority trainees into science. In addition to meeting with leadership at the Atlanta VA, Dr. Shepherd interviewed with representatives from ORD leadership for vetting and additional discussions of program structure and plans. ORD CRS funding was used to support a portion of Dr. Shepherd's professional effort as CRS director and to provide part-time administrative assistant effort to support CRS activities.

An array of activities was devised to establish a recruitment pathway. To enhance awareness of the program among Morehouse faculty and trainees, the CRS director, with input from the executive associate directors, developed a descriptive presentation of the VA CDA program that was delivered to targeted department chairs and center directors at Morehouse. The CRS director also held face-to-face meetings with these Morehouse leaders to re-emphasize the program and renew awareness over time. The CRS director solicits feedback to further improve recruitment efforts and penetration of targeted information to all eligible candidates. Printed informational and promotional materials describing the VA CDA program were prepared for display and distribution to trainee candidates.

Ongoing efforts will develop a Morehouse website that includes descriptions of the VA CDA program and VA research in general, contact information for the CRS director, as well as other key individuals, with links to websites describing ongoing VA research activities that could sponsor trainees. The website will also feature a VA or Morehouse investigator once per quarter who could potentially serve as a VA CDA mentor. Previously featured mentors would be archived on the site to develop a “library” of eligible mentors and program proponents. The website will also include descriptions of current and former trainees, highlighting their research programs, their accomplishments, and their current positions.

Given that funding for the Atlanta VA–MSM CRS began in spring 2020, initial plans for face-to-face events were interrupted by the COVD-19 pandemic and restrictions established at both institutions. For example, initial plans included an annual day-long joint scientific meeting with Atlanta VA—Morehouse investigators and their trainees. The location of the conference will be rotated between sites, include presentations by local experts and trainees, award top trainee presenters, and include poster and oral sessions with coffee breaks, lunch, and distribution of CRS program information materials. The goals of these meetings will be to promote the VA CDA program and to grow collaborations between Atlanta VA and MSM trainees and investigators. The feasibility of such a program was realized in part by the Atlanta VA Research Day celebration in May 2022 when >170 faculty and trainees from VA, MSM, and Emory gathered for a day-long scientific program with featured speakers, posters, and networking opportunities.

The CRS will further facilitate the success of recruited candidates by forming a mentorship committee for each trainee comprising faculty from the Atlanta VA and MSM to promote expanded training opportunities and to facilitate new collaborations among faculty. Strategic efforts will be made to include faculty from both institutions to promote collaborative teams. Each awardee's mentorship committee will meet every 6 months to ensure that awardees are making adequate progress and to address barriers promptly. These committee meetings will also ensure that candidates are on schedule for submitting for the next level of VA funding (e.g., Merit Review Award) well before expiration of the CDA funding. Atlanta VA Research Office administrative personnel will help candidates navigate the VA grant submission process and provide examples of successful applications.

The Atlanta VA Research Office has also recently begun a quarterly meeting that includes all VA CDA awardees, applicants, and those interested in applying. The goal of these meetings is to build community among early-career investigators at VA, to promote peer-to-peer mentoring, sharing of best practices, and scientific interchange. CRS-identified HBCU candidates will be included in these meetings to further promote integration of Morehouse trainees into the Atlanta VA scientific community. During these seminars, CDA awardees will give brief scientific seminars (e.g., 30 min in duration) on a rotating basis to hone their scientific presentation skills. The remainder of the session will include presentations from senior mentoring faculty discussing topics relevant to career development, VA funding mechanisms, and research opportunities.

These recurring meetings will provide opportunities for the CRS director to share updates and announcements with awardees. Selected sessions might include presentations from invited guests (e.g., representatives from ORD), local centers, and local VA Research Office representatives addressing issues related to biosafety, animal care, human subjects research, or budget management. Combined with the annual scientific meeting described earlier, these activities will provide familiarity and community among Atlanta VA and MSM investigators. Additional training opportunities will include chances for CDA awardees to review new CDA applications to provide critical comments and relevant experience to enhance their awareness of grantsmanship issues and to promote further development of their grant reviewing skills.

## Program Oversight

The weekly operations of the CRS are reviewed and discussed during monthly virtual meetings with the CRS director, administrative assistant, and the two executive associate directors present. The eligibility of individual candidates is reviewed, discussed, and strategies for candidate recruitment are developed. The CRS also submits an annual progress report to VA ORD, and ORD personnel periodically join monthly CRS meetings for questions and updates. As an additional local mechanism for oversight and guidance, a CRS Advisory Committee was established. Composed of three faculty leaders from the Atlanta VA and three faculty leaders from MSM, the CRS Advisory Committee assists with oversight and guidance of the program.

The members of the Advisory Committee were selected based on their knowledge of research and related programs at their respective institutions, and all enthusiastically agreed to participate. The executive associate directors also participate in the CRS Advisory Committee. The CRS director convenes the Advisory Committee twice yearly to report on progress and to identify and resolve any barriers to recruitment. These discussions are designed to promote and ensure achievement of target recruiting goals and to facilitate communication between interested faculties of the two institutions related to career development training. The Advisory Committee can also be convened as needed to address any *ad hoc* or urgent issues.

## Challenges and Future Opportunities

The CRS held discussions with a number of interested candidates from HBCUs in the Atlanta University Consortium (including Morehouse College, Spelman College, Clark Atlanta University, and MSM). Not infrequently, we discovered that potential candidates had CDA eligibility issues most often related to level of training or time since conferral of terminal degree. In select cases, more experienced and senior candidates with existing research accomplishments were encouraged to consider VA Merit Review Award funding mechanisms. In addition, several qualified candidates elected to pursue alternative professional pathways outside of academic medicine, including careers in industry.

Despite these challenges, the CRS has exerted a palpable and positive impact on the research affiliation between MSM and the Atlanta VA. We believe these efforts to build a pipeline for recruiting HBCU candidates to VA research as described earlier has launched a cultural shift in our organizations' collaborative view of career development opportunities for talented HBCU professional candidates. CRS support also enabled a successful competitive 2-month summer VA research experience for HBCU students in 2022. This program, led by Drs. Olamide Alabi and Ashley Scales, selected four MSM professional students to spend 2 months working with a senior VA mentor and their team on a funded VA research project. The CRS provided the students' stipends while the Atlanta VA Research Administration Office addressed the onboarding of the students and organized a weekly seminar targeted to their career interests.

Students who completed the program provided extremely positive reviews that motivated plans to double the size of the program planned for summer 2023. These activities illustrate how the CRS can support creative and novel approaches to increase awareness of VA Research careers among HBCU trainees. Continued growth of these programs will benefit from proportionate growth in the administrative structure supporting the trainees and their recruitment, and from systems to adequately track the successes of trainees as they advance in their careers. Going forward, we envision a growing population of successful Merit Review-funded MSM faculty at VA who serve as mentors and powerful examples of professional and career accomplishment for HBCU trainees within VA.

## Abbreviations Used

CDA: Career Development Awards
CRS: Core Recruiting Sites
HBCUs: Historically Black Colleges and Universities
MD: Doctor of Medicine
MSM: Morehouse School of Medicine
ORD: Office of Research and Development
VA: Department of Veterans Affairs
